# Double-Loaded Doxorubicin/Resveratrol Polymeric Micelles Providing Low Toxicity on Cardiac Cells and Enhanced Cytotoxicity on Lymphoma Cells

**DOI:** 10.3390/pharmaceutics15041287

**Published:** 2023-04-19

**Authors:** Lyubomira Radeva, Yordan Yordanov, Ivanka Spassova, Daniela Kovacheva, Virginia Tzankova, Krassimira Yoncheva

**Affiliations:** 1 Faculty of Pharmacy, Medical University of Sofia, 1000 Sofia, Bulgaria; 2 Institute of General and Inorganic Chemistry, Bulgarian Academy of Sciences, 1113 Sofia, Bulgaria

**Keywords:** polymeric micelles, double drug loading, doxorubicin, resveratrol, cardioprotection

## Abstract

The anthracycline antibiotic doxorubicin is a well-known antitumour agent, however its cardiotoxicity is a significant obstacle to therapy. The aim of the present study was to improve the safety of doxorubicin through its simultaneous encapsulation with a cardioprotective agent (resveratrol) in Pluronic micelles. The formation and double-loading of the micelles was performed via the film hydration method. Infrared spectroscopy proved the successful incorporation of both drugs. X-ray diffraction analyses revealed that resveratrol was loaded in the core, whereas doxorubicin was included in the shell. The double-loaded micelles were characterised by a small diameter (26 nm) and narrow size distribution, which is beneficial for enhanced permeability and retention effects. The in vitro dissolution tests showed that the release of doxorubicin depended on the pH of the medium and was faster than that of resveratrol. In vitro studies on cardioblasts showed the opportunity to reduce the cytotoxicity of doxorubicin through the presence of resveratrol in double-loaded micelles. Higher cardioprotection was observed when the cells were treated with the double-loaded micelles compared with referent solutions with equal concentrations of both drugs. In parallel, treatments of L5178 lymphoma cells with the double-loaded micelles revealed that the cytotoxic effect of doxorubicin was enhanced. Thus, the study demonstrated that the simultaneous delivery of doxorubicin and resveratrol via the micellar system enabled the cytotoxicity of doxorubicin in lymphoma cells and lowered its cardiotoxicity in cardiac cells.

## 1. Introduction

Polymeric micelles are commonly used carriers for various drugs due to their considerable advantages. They are characterised by a core–shell structure that allows for the high loading of hydrophobic drugs and improvement of their solubility. Moreover, their small size enhances some pharmacologically relevant processes. The nanoscale size and the hydrophilic shell of the micelles (e.g., based on poly(ethylene oxide) (PEO)) hinder phagocytosis and prolong the circulation time of micelles after systemic injection. The small size is also a prerequisite for the enhanced permeability and retention (EPR) effect to occur, which results in accumulation in tumours and inflamed tissues [1,2,3,4]. Furthermore, these systems are amenable to double loading, which may provide synergistic effects, or combining drugs with different pharmacological activities [5,6]. For instance, Scarano et al. accomplished a high synergistic anticancer effect in human ovarian cancer cells (A2780) by simultaneously encapsulating curcumin and oxoplatin in micelles based on polycaprolactone, polyethylene glycol and amine-bearing polymers [7]. Similarly, Duong and Yung synthesized double-loaded with doxorubicin and paclitaxel poly(ethylene glycol)-poly(d,l-lactide-co-glycolide)-folate-TAT peptide-based micelles, which showed a significant decrease in IC_50_ values in human carcinoma KB cell lines [8]. Double loading of highly toxic substances (e.g., anticancer drugs) with antioxidants is a promising approach to decrease their toxicity to healthy tissues and cells. Riedel et al. simultaneously encapsulated paclitaxel and curcumin in Soluplus:TPGS mixed micelles and observed a reduced paclitaxel-induced neurotoxic effect, due to the antioxidant properties of curcumin [9]. In addition, by obtaining micellar nanocomplexes between hyaluronic acid-epigallocatechin-3-O-gallate conjugate and cisplatin, the off-target organ toxicity of the anticancer drug was reduced while the antitumour activity was enhanced [10].

Doxorubicin (Dox) is an anthracycline antibiotic produced by different strains of Streptomyces bacteria and possesses a broad-spectrum anticancer activity. Its mechanism of action is related to interference with the biosynthesis of DNA by affecting topoisomerase II. The main limitations of doxorubicin are that it is considerably non-selective and exerts quite pronounced cardiotoxic effects. Its cardiotoxicity is associated with increased levels of reactive oxygen species (ROS), lipid peroxidation, decreased levels of antioxidants, apoptosis caused by caspase activation and internucleosomal DNA degradation, intracellular calcium dysregulation and subsequent myofibrillar deterioration [11,12]. A possible solution for this problem could be the simultaneous loading of doxorubicin and substances that reduce its toxicity into nanosized micelles. Polymeric micelles offer a suitable drug delivery system for such double loading. Substances that could reduce the levels of reactive oxygen species and lipid peroxidation provoked by doxorubicin are natural antioxidants, of which polyphenols are of the greatest interest. Ma et al. encapsulated curcumin and doxorubicin in hyaluronic acid-vitamin E succinate (HA-VES) graft polymer micelles [13]. In vitro tests on resistant MCF-7/Adr cells and in vivo tests on tumour-bearing mice showed that double-loaded micelles had better cytotoxicity, apoptotic effect, tumour accumulation, inhibition of tumour growth and, most significantly, protective effect against doxorubicin-induced cardiotoxicity. Another study reported the co-encapsulation of doxorubicin and curcumin in monomethoxy poly(ethylene glycol)–poly(ε-caprolactone)–N-t-butoxycarbonyl-phenylalanine micelles. The formulated double-loaded micelles provided cardioprotection in H9c2 cardioblast cells and, at the same time, inhibition of tumour growth in tumour-bearing mice [14]. Similar results were achieved by another group, who simultaneously loaded quercetin and doxorubicin in methoxy poly(ethylene glycol)-poly(D, L-lactide-co-glycolide) micelles [15]. 

Resveratrol is a polyphenol that possesses various remarkable activities; among them, anti-inflammatory, anticancer, antiangiogenic, antibacterial, neuroprotective and cardioprotective effects were well documented [16,17,18,19]. The mechanism of its cardioprotective action was considered to be related to suppressed platelet aggregation, vasodilatation, reduction of reactive oxygen species (ROS), prevention of oxidation of low-density lipoprotein (LDL) and modulation of important signaling pathways [20,21]. It is important to note that the toxic effects of anticancer agents on healthy cells could be reduced by resveratrol. For instance, Gu et al. reported that oxidative stress, apoptosis, autophagy and fibrosis induced by doxorubicin were alleviated by resveratrol [22]. At the same time, various studies revealed that resveratrol stimulated the apoptosis of tumour cells, modulated vascular endothelial growth factor and inhibited angiogenesis, blocked tumour growth and migration and also inhibited the cyclooxygenase (COX) activity [23,24]. An in vitro study on MCF-7 and MDA-MB-231 cells and in vivo on Ehrlich ascites carcinoma (EAC) cell-bearing mice showed that an application of resveratrol and doxorubicin resulted in a synergistic cytotoxic effect [25]. Another study reported that the treatment with both drugs on the same carcinoma-bearing mice model increased the survival rate and resulted in the absence of muscle-fiber fragmentation in the heart tissue [26]. However, the low water solubility, problematic stability, fast metabolism and poor bioavailability hindered its application [27,28]. Thus, the formulation of a resveratrol and cytotoxic drug in nanoparticles could overcome these limitations. Carslon et al. encapsulated resveratrol and curcumin in Pluronic micelles and discovered that they potentiated the cytotoxic effect of doxorubicin in ovarian cancer cells (SKOV-3) and alleviated toxicity in cardiomyocytes (H9c2) [29]. In vivo studies showed that double-loaded (resveratrol and curcumin) polymeric micelles simultaneously administered with non-encapsulated doxorubicin exerted a synergetic effect on the cancer-bearing mice and a cardioprotective effect on the healthy animals [30]. Thus, a new strategy could be the simultaneous encapsulation of the polyphenol and doxorubicin in a single nanoparticle system that would overcome the need for their separated administration. 

Various copolymers may be used for the preparation of micellar drug delivery systems, but the most commonly applied are the triblock copolymers of poly(propylene oxide) and poly(ethylene oxide), known as Pluronics. It is well known that these copolymers can inhibit the P-glycoprotein (P-gp) pump, which may facilitate resolving the problem of multiple drug resistance (MDR). Wei et al. observed that P-gp inhibition by Pluronic copolymers is associated with the reduction of intracellular adenosine triphosphate (ATP) [31]. Encapsulation in Pluronic micelles has been reported to successfully improve the unfavourable physicochemical and biopharmaceutical characteristics of various drugs, in particular paclitaxel [32,33], lacidipine [34], isoliquiritigenin [35], niclosamide [36], sorafenib and verteporfin [37], curcumin [38,39], and cannabidiol [40]. 

The aim of the present study was to prepare nanoscale micelles, providing the opportunity for the simultaneous encapsulation of doxorubicin and resveratrol in a combined drug delivery system. The novelty of such a system would be the easy administration of both drugs and the protection of cardiac cells from doxorubicin toxicity without affecting its cytotoxicity on tumour cells. To achieve this, double-loaded mixed Pluronic micelles with doxorubicin and resveratrol were developed. The protective capacity of the double-loaded micelles against doxorubicin toxicity was studied in rat cardioblasts, whereas the cytotoxic effects were evaluated in lymphoma L5178 cells. 

## 2. Materials and Methods

### 2.1. Materials

Pluronic^®^ P 123 (PEO_20_PPO_70_PEO_20_) and Pluronic^®^ F 127 (PEO_101_PPO_56_PEO_101_) were supplied by BASF, Ludwigshafen, Germany. Doxorubicin hydrochloride and trans-resveratrol were purchased from Sigma Chemical Co. (Schnelldorf, Germany). Neutral red was supplied by Thermo Fisher Scientific (Waltham, MA, USA). Dulbecco’s Modified Eagle’s Medium, McCoy’s 5A Medium, foetal bovine serum (FBS) and L-glutamine were purchased from Sigma-Aldrich (Merck KGaA, Darmstadt, Germany). The cardioblast cell line H9c2 was obtained from the European Collection of Cell Cultures (ECACC, Salisbury, UK). The mouse lymphoma cell line L5178 was donated by Dr. M. M. Gottesman (National Cancer Institute, Bethesda, MD, USA).

### 2.2. Determination of Compatibility between Both Drugs and Hydrophobic Block of the Copolymers

For the determination of compatibility between both drugs and the hydrophobic block of the copolymers (poly(propylene oxide), PPO), the Flory–Huggins parameter χ_sp_ was calculated by the following equation:

(1) $$\chi_{\mathrm{sp}}=\frac{V_{s}{\left(\delta_{s}-\delta_{p}\right)}^{2}\;}{\mathrm{RV}}$$

where V_s_ is the molar volume of the drugs, δ_s_ and δ_p_ are the solubility parameters of the drugs and the core-forming polymer, R is the gas constant and T is the Kelvin temperature [41]. The solubility parameters of each drug and PPO block were calculated using the contribution of the chemical groups in the molecules to their cohesive energy [42]. 

### 2.3. Preparation of Double-Loaded Micelles

The polymeric micelles were prepared via the film hydration method. For this purpose, 4 mg of doxorubicin was dissolved in 6 mL of methanol. Separately, different amounts of resveratrol were dissolved in a methanol solution (4 mL) containing two Pluronic copolymers, in particular 20 mg of P123 and 20 mg of F127. After that, the solution of doxorubicin was added to the polymeric solution and the solvent was evaporated under stirring (700 rpm) at room temperature. Finally, the resulting micelles were redispersed in 4 mL of purified water and the dispersion was filtered (0.2 µm). The concentrations of doxorubicin and resveratrol in the rinsed filter fractions were determined by UV spectrophotometry at 480 nm and 306 nm, respectively (Thermo Fisher Scientific, Waltham, MA, USA). The rinsing was sequentially performed with purified water and a hydroalcoholic mixture containing 50% ethanol. The concentrations were calculated using standard curves obtained in the range of 10–80 µg/mL (r > 0.9991) for doxorubicin and 2–10 µg/mL (r > 0.9996) for resveratrol. The standard curve of doxorubicin was prepared in purified water and that of resveratrol in water containing 50% ethanol. The following equations were applied for the determination of the loading degree (LD) and encapsulation efficiency (EE):

LD = (Total amount of drug − Non-loaded drug)/Volume of drug loaded micellar dispersion(2)

EE = (Total amount of drug − Non-loaded drug)/Total amount of drug(3)

### 2.4. Characterisation of the Micelles

The determination of the average diameter and polydispersity index of the micelles was carried out by dynamic light scattering (DLS) using Zetasizer NanoBrook 90Plus PALS (Brookhaven Instruments Corporation, Holtsville, NY, USA), equipped with a 35-mW red diode laser (λ = 640 nm) at a scattering angle of 90°. The zeta potential of the micelles was determined through a phase analysis light scattering (PALS) method at a scattering angle of 15°.

Powder X-ray diffraction patterns of empty Pluronic F127/P123 micelles, resveratrol, doxorubicin and the double-loaded micelles were collected in the 10–80° 2 theta range using a Bruker D8Advance diffractometer (Bruker Corporation, Billerica, MA, USA) equipped with Cu-tube (λ = 1.5418 Å) and a LynxEye detector. The degree of crystallinity was determined with the Topas 4.2 program, using the standard procedure described in the Topas tutorial [43].

Infrared spectra (IR) of doxorubicin, resveratrol and empty and double-loaded micelles in KBr were collected using a Thermo Nicolet Avatar 360 FTIR spectrometer (Thermo Fisher Scientific, Waltham, MA, USA), with a resolution of 2 cm^−1^.

### 2.5. In Vitro Release of Doxorubicin and Resveratrol from the Micelles

An in vitro release study was conducted using a dialysis method in two buffer media (pH-values 7.4 and 5.0) containing 10% ethanol. An exact volume of micellar dispersion (3.2 mL containing 2.88 mg resveratrol and 2.87 mg doxorubicin) was introduced in a membrane (10,000 MWCO, Spectrum Labs), which was placed in 80 mL of acceptor phase (phosphate or citrate buffer). The test was carried out under gentle stirring at a water bath temperature of 37 °C (IKA Labortechnik HS-B20, Staufen, Germany). Samples of 6 mL were withdrawn from the acceptor phase at predetermined intervals for the determination of the concentration of the released doxorubicin and resveratrol. The equivalent volume of a fresh buffer solution was added back to maintain sink conditions. The concentrations of the released doxorubicin and resveratrol were determined by UV spectrophotometry at 480 nm and 306 nm, respectively (Thermo Fisher Scientific, Waltham, MA, USA).

The mechanism of drug release was evaluated via fitting the data to a Korsmeyer–Peppas model [44]. The data used for the analyses were until the point of 60% release of the drugs. The determination of correlation and diffusional exponent (n) was performed by applying the equation:

M_t_/M_∞_ = kt^n^ (4)

where M_t_/M_∞_ is the accumulated fraction of drug release, t is the respective time and k is the release rate constant.

### 2.6. Cell Culturing and In Vitro Cell Viability Studies

The viability studies on rat cardioblast cells (H9c2) were performed via neutral red assay [45]. First, the cells were seeded in 96-well plates at a cell density of 5 × 10^3^ and incubated overnight at 37 °C, 5% CO_2_ and high humidity (Esco CelCulture^®^ CO_2_ Incubator, CCL-170B-8-IVF, Esco Micro Pte. Ltd., Singapore). After 24 h of incubation, the cells were treated with an aqueous solution of pure doxorubicin (0.01–80 μM), the developed double-loaded micelles and referent hydroalcoholic solutions containing doxorubicin and resveratrol. The concentrations of doxorubicin in the micelles and referent solutions were 0.25 or 5 μM; that of resveratrol ranged between 0.3 and 26 μM. Further, a 100 µL solution of neutral red in cell culture (40 µg/mL) was added to each well and the plates were incubated for 3 h at 37 °C. Then, the cells were washed with phosphate-buffered saline (PBS), and 100 µL of a destaining solution per well was added. The plates were rapidly shaken for 10 min and the optical density was measured in a Synergy 2 multiplate reader (BioTek Instruments, Inc., Highland Park, Winooski, VT, USA) at 540 nm.

The effects of the samples were also evaluated in mouse lymphoma L5178 cells. The cells were seeded in 96-well plates at a cell density of 1 × 10^4^ and incubated under the same conditions. After 24 h, the cells were treated with double-loaded micelles or the corresponding hydroalcoholic solutions of the non-encapsulated doxorubicin and resveratrol in the same concentrations as described above. An Alamar blue assay was used for the evaluation of lymphoma cell viability [46]. A resazurin solution in PBS was added to each well (44 µM final concentration). The fluorescence at 540 nm was measured in the Synergy 2 multiplate reader immediately after resazurin addition for baseline fluorescence evaluation and after 3 h of incubation for viability evaluation.

### 2.7. Statistical Analysis

The results were expressed as mean values ± SD (*n* = 9). GraphPad Prism 8 Software (Dotmatics, San Diego, CA, USA) was used for the statistical analysis. One-way ANOVA with Dunnett’s multiple comparison post-test was applied in order to compare the different groups of cells treated with pure doxorubicin, double-loaded micelles and standard solutions of both drugs. Furthermore, multiple *t*-tests with Holm–Sidak correction were used to compare corresponding groups of cells treated with either double-loaded micelles or hydroalcoholic solutions of drugs. In order to assess the strength of observed tendencies, effect sizes with corresponding confidence intervals were calculated by means of an effectsize package in R [47,48].

## 3. Results

Double-loaded nanosystems are an alternative approach for the simultaneous delivery of two active molecules. In the present study, the attempts were directed to the double loading of doxorubicin and resveratrol in Pluronic micelles. Resveratrol was selected as an antioxidant agent because of its potential to alleviate oxidative stress in cardiomyocytes, to counter apoptosis and to relieve cardiac fibrosis induced by doxorubicin [19,22]. Thus, the purpose of the double loading was to ensure that, upon the simultaneous delivery of both drugs, the released resveratrol would protect cardiac cells from doxorubicin toxicity and the cytotoxicity of doxorubicin in tumour cells would be maintained.

**Figure 1:**
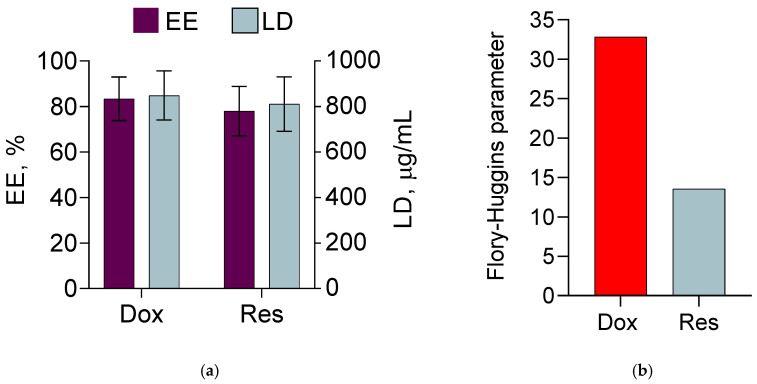
Encapsulation efficiency (EE) and loading degree (LD) in the double-loaded micelles (**a**) and Flory–Huggins parameter calculated for doxorubicin (Dox) and resveratrol (Res) with respect to PPO block (**b**).

**Figure 2:**
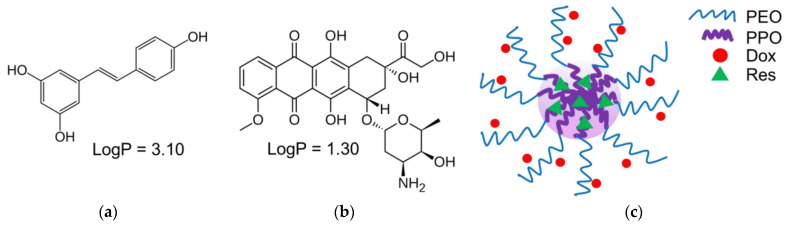
Structures and partition coefficients of resveratrol (**a**) and doxorubicin (**b**) and schematic presentation of their loading in the micelles (**c**).

The micelles were formulated with two types of Pluronic copolymers, particularly Pluronic P123 and Pluronic F127. The combination of Pluronics with different hydrophilic–lipophilic balance (HLB) values (8 and 22, respectively) was believed to ensure an efficient loading of both drugs. The micelles were prepared at a ratio 1:1 (wt/wt) between the copolymers, since a previous study showed their high stability [40]. The double drug loading was performed via the film hydration method. Methanol was selected as a solvent because the copolymers and both drugs could be dissolved in it. The drug loading and encapsulation efficiency are presented in Figure 1a. The results revealed high values for loading and efficiency for both drugs (83.4% for doxorubicin and 78% for resveratrol), independent of having different affinity. In particular, the calculation of the Flory–Huggins parameter showed that the affinity of resveratrol to the PPO core of the micelles was higher (lower value) than that of doxorubicin (Figure 1b). These results correlated with the partition coefficients of both drugs that indicate the stronger lipophilic properties of resveratrol (logP of 3.1) and the more hydrophilic properties of doxorubicin (logP of 1.30) [27,49]. Thus, taking into consideration both the values of the Flory–Huggins parameter (Figure 1b) and the partition coefficients (Figure 2a,b), we suggested that resveratrol was most probably loaded into the micellar core, whereas doxorubicin was located in the shell (Figure 2c).

**Figure 3:**
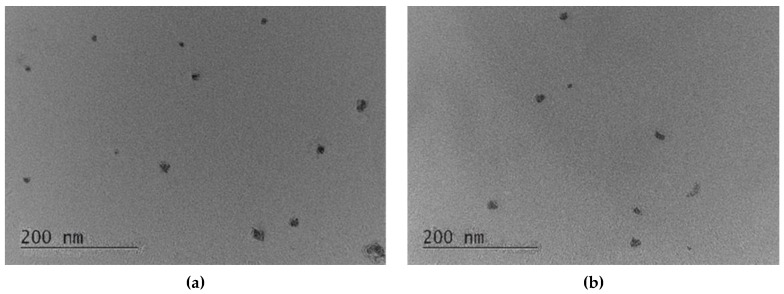
Transmission electron microscopy of empty (**a**) and doxorubicin/resveratrol-loaded micelles (**b**).

**Figure 4:**
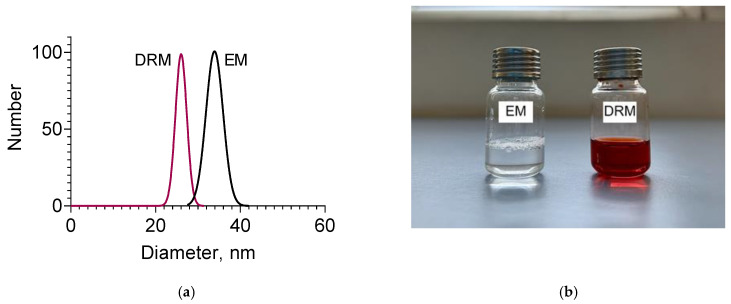
Mean diameters (**a**) and digital image (**b**) of empty (EM) and double-loaded doxorubicin/resveratrol micelles (DRM).

Transmission electron microscopy showed a spherical shape and a size smaller than 200 nm of both empty and double-loaded micelles (Figure 3a,b). DLS measurements confirmed the small diameters of the micelles and showed that the empty micelles were slightly larger than the drug-loaded micelles (Figure 4 and Table 1). The smaller diameter of the loaded micelles could be explained with an enhanced cohesive force of hydrophobic interaction between resveratrol and PPO chains. A similar decrease in the size of the drug-loaded micelles was observed upon docetaxel loading in mixed monomethylol poly(ethylene glycol)-poly(D,L-lactic acid) (MPP)/D-α-tocopheryl polyethylene glycol 1000 succinate micelles [50]. As seen, the micellar dispersions were characterised by narrow size distribution. These results suggested that the developed micelles would be appropriate for future usage as a double-loaded drug delivery system in chemotherapy. The zeta potential of the empty micelles was slightly negative, which was consistent with the non-ionic properties of the Pluronic copolymers (Table 1). However, the zeta potential of the double-loaded micelles was slightly positive (Table 1). This change could be considered as an indication that the amino groups of doxorubicin, which were located close to the surface of the micelles (Figure 2c), contributed to the positive charge.

**Figure 5:**
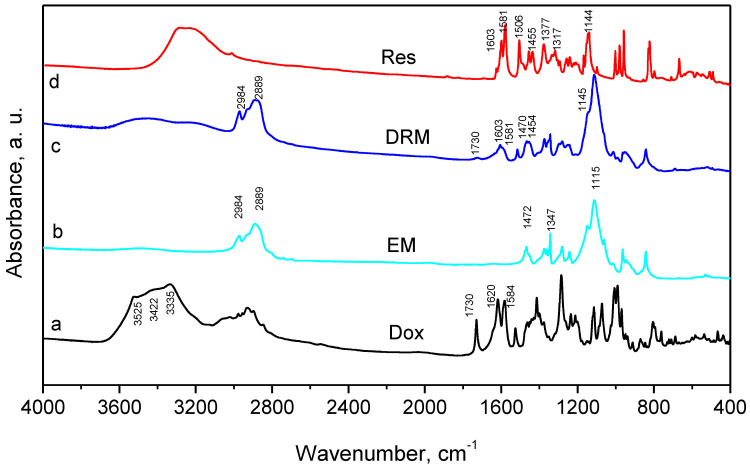
FTIR spectra of doxorubicin (Dox), resveratrol (Res), empty (EM) and double-loaded micelles (DRM).

The incorporation of both drugs was studied by comparison of IR spectra of pure drugs and double-loaded micelles (Figure 5). The FTIR spectra of the empty micelles (EM) presented overlapping C-O-C stretching vibrations in the ~1200–1000 cm^−1^ range, characterised by a triplet of intense bands at 1059, 1115 and 1142 cm^−1^ and C–H stretching vibrations of PEO fragments at 2984 and 2889 cm^−1^. The band at 1472 cm^−1^ was ascribed to a methylene scissoring mode and the line at 1347 cm^−1^ could be attributed to an in-plane O-H bend [51]. The spectra of pure doxorubicin completely coincided with those in our previous paper [52]. The spectra of pure resveratrol presented bands at 3200 cm^−1^, attributed to the O-H stretching mode, three characteristic bands at 1603, 1581 and 1377 cm^−1^, assigned to C=O stretching vibration of the phenol rings and a C=C olefinic bond [53]. The bands at 1506, 1455 and 1317 cm^−1^ could be due to the bending vibrations of C-H bonds, respectively [54]. The spectra of the double-loaded micelles evidenced the presence of doxorubicin (with its characteristic bands at 1730 cm^−1^ (attributed to C=O) and at 1620 cm^−1^ and 1584 cm^−1^ (ascribed to the phenol ring)), as well as the presence of resveratrol in the investigated micelles, exhibiting the lines at 1603 cm^−1^ and 1581 cm^−1^.

**Figure 6:**
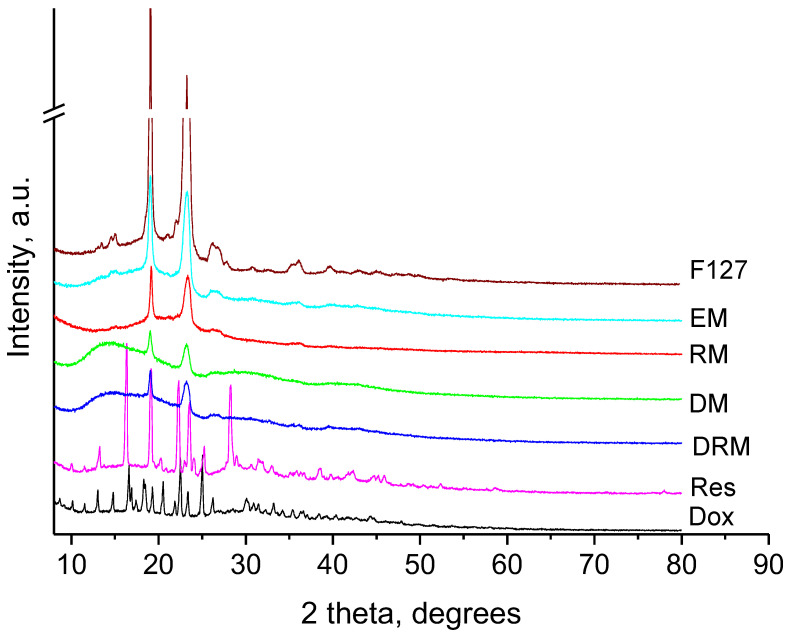
Powder XRD patterns of Pluronic F127 (F127), empty micelles (EM), resveratrol (Res), doxorubicin (Dox), single resveratrol-loaded micelles (RM), single doxorubicin-loaded micelles (DM) and the double-loaded micelles (DRM).

Further, in an attempt to evaluate the localisation of resveratrol and doxorubicin in the micelles, X-ray diffraction (XRD) analyses were performed. The collected XRD patterns are presented in Figure 6. The presence of sharp peaks in the doxorubicin XRD pattern showed its crystalline nature [52]. The high crystalline state was also observed for the pure resveratrol. The refined unit cell parameters were close to those reported in the literature [55]. It is worth mentioning that the loading of resveratrol and doxorubicin resulted in the total deterioration of their crystal structures, since no peaks belonging to either compound were found in the XRD patterns of the loaded samples. The XRD patterns of Pluronics comprised a crystalline part originating from the PEO fragments and an amorphous part corresponding to the PPO blocks; this is why its structure can be regarded as semicrystalline [56]. The degree of crystallinity reflected the ratio of these two units building the particular Pluronics. In the present case, the crystallinity of the empty Pluronic F127/P123 micelles was 32%, as the Pluronic F127 with 54% crystallinity was mixed with Pluronic P123 with 12% crystallinity. Single loading of resveratrol in the micelles led to the decrease in the degree of crystallinity to 21%, while the single loading of doxorubicin in the micelles lowered the degree of crystallinity to 16%. This result advocated the hypothesis for the localisation of resveratrol and doxorubicin in the different blocks of the micelles’ structures. It seems that the resveratrol tended to piece on the hydrophobic PPO part (core) and the doxorubicin preferably connected to the hydrophilic PEO part (shell) of the micelles. The double-loaded micelles (DRM) showed 14% degree of crystallinity, confirming the dominating interaction of doxorubicin with PEO crystalline blocks.

**Figure 7:**
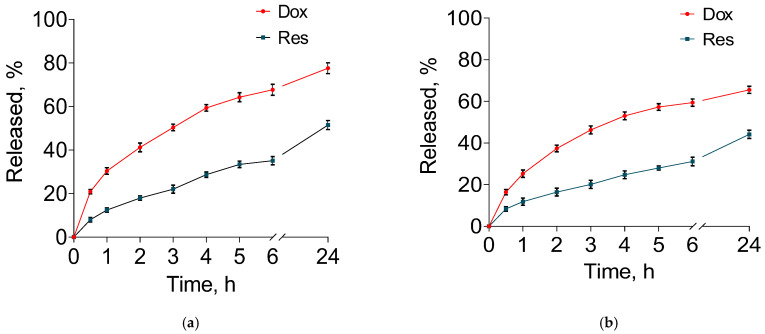
In vitro release profiles of doxorubicin and resveratrol from the double-loaded micelles in buffers with pH = 5 (**a**) and pH = 7.4 (**b**).

In vitro tests of drug release were performed in two different media with pH 7.4 and 5.0 (Figure 7a,b). In both release media, there was a faster release of doxorubicin compared with resveratrol. In particular, between 60 and 70% doxorubicin was released for 6 h in both media compared with 30 to 35% resveratrol for the same time. Furthermore, the burst release was also more pronounced for doxorubicin. The latter could be due to the surface location of doxorubicin as suggested by the zeta potential measurements and XRD analyses. Moreover, in the acidic medium, a tendency for a faster release of doxorubicin was observed compared with that in the alkaline medium (Figure 7a). It was considered that the solubility of doxorubicin increased in the acidic medium due to its basic character [57,58,59]. Zhang et al. discovered that the doxorubicin’s solubility was 0.51 mg/mL in a slightly alkaline medium (pH = 7.4) and 1.04 mg/mL in a slightly acidic medium (pH = 5.0) [60]. Thus, the hydrophilicity of doxorubicin in the slightly acidic medium determined the tendency for its faster release. In comparison, resveratrol was highly hydrophobic and its affinity to the micellar core (Figure 1b) slowed down the release rate. Similarly, a simultaneous release of doxorubicin and paclitaxel from double-loaded functionalised PLGA-PEG micelles showed a slower release of paclitaxel, which is a more hydrophobic drug [8].

To gain insight into the mechanism of drug release, the data were fitted to a Korsmeyer–Peppas model. According to the model, values of the diffusional exponent (n) between 0.43 and 0.85 (for spheres) indicated a non-Fickian diffusion. Table 2 presents the values for the correlation coefficient (r^2^) and the diffusional exponent (n). It was found that there was a well-pronounced correlation to the model for both drugs. In the case of doxorubicin, the values of the exponent were 0.497 and 0.530 for the release in an acid and a neutral medium, respectively. In the case of resveratrol, the values of the exponent were lower, in particular 0.443 and 0.432, respectively. Thus, the data suggested that the release of the drugs occurred by non-Fickian diffusion, including diffusion and polymer relaxation processes.

**Figure 8:**
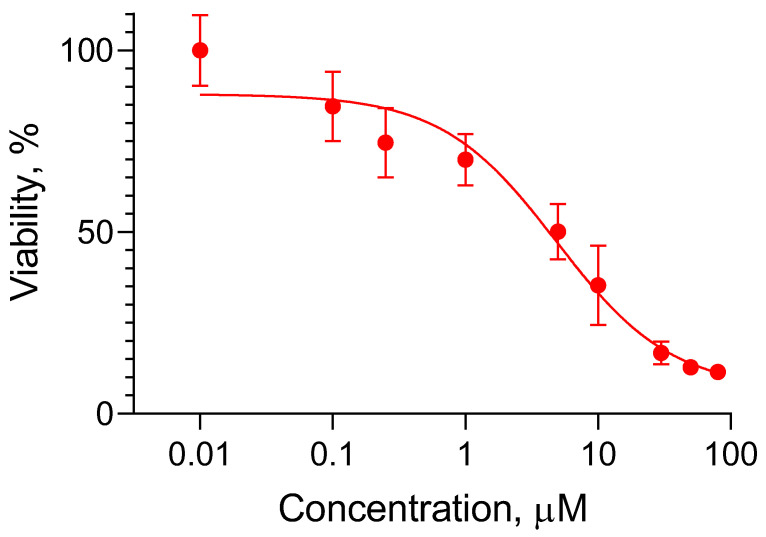
In vitro cytotoxic effect of pure doxorubicin in H9c2 cells after 24 h treatment.

**Figure 9:**
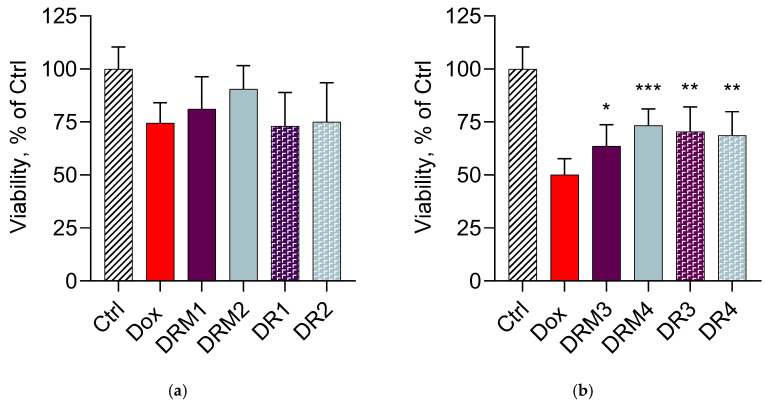
In vitro cell viability of cardioblasts (H9c2) after treatment with the double-loaded doxorubicin/resveratrol micelles (DRM) and referent hydroalcoholic solutions of both drugs (DR). Ctrl—non-treated cells; DRM1 (double-loaded micelles) and DR1 (standard solution) containing 0.25 µM Dox and 1.3 µM Res (**a**); DRM2 (double-loaded micelles) and DR2 (standard solution) containing 0.25 µM Dox and 0.3 µM Res (**a**); DRM3 (double-loaded micelles) and DR3 (standard solution) containing 5 µM Dox and 26 µM Res (**b**); DRM4 (double-loaded micelles) and DR4 (standard solution) containing 5 µM Dox and 6 µM Res (**b**); * *p* < 0.05; ** *p* < 0.01; *** *p* < 0.001 vs. doxorubicin group.

Our next studies aimed to evaluate the capacity of the double-loaded micelles to decrease the cardiotoxicity of doxorubicin in vitro. First, the examination of cytotoxicity of doxorubicin on cardioblasts (H9c2 cells) showed a dose-dependent effect (Figure 8). The obtained IC_25_ and IC_50_ of the pure drug were about 0.25 µM and 5 µM, respectively. Other research groups have reported similar values of IC_50_ in H9c2 cells at the 24th h of treatment, namely 4.3 µM [29], 7.045 µM [61] and approximately 2.5 µM [62]. Thus, our assumption was that the double encapsulation of doxorubicin and resveratrol in Pluronic F127/P123 micelles would decrease the cardiotoxicity of doxorubicin. In order to prove this, the cardioblasts were treated with double-loaded micelles containing doxorubicin in concentrations corresponding to its IC_25_ and IC_50_. In parallel, the cardioblasts were also treated with standard solutions of doxorubicin and resveratrol in the same concentrations (Figure 9a,b). The incubation of the cells with a combination of 0.25 µM doxorubicin and two concentrations of resveratrol is presented in Figure 9a. As seen, a more pronounced tendency for cardioprotection was observed with the double-loaded micelles. The referent hydroalcoholic solutions of doxorubicin and resveratrol had no protective effect and this result indicated the importance of the nanomicellar system. Most probably, the micellar system contributed to the higher protection due to the ability of enhancing cell uptake and also of inhibiting P-gp efflux [63,64]. Interestingly, a higher protection (Cohen’s d 1.02; 95% confidence interval [0.02, 1.99], size of effect: large [65]) was registered with the micelles containing a lower concentration of resveratrol (0.3 µM). A possible explanation for this phenomenon could be the fact that, depending on the concentration and different cell types, resveratrol could exert cytotoxic and prooxidant activity [66]. The examination of the higher cytotoxic concentration of doxorubicin (5 µM) showed significant cardioprotective effects of both the micelles and the referent solutions (Figure 9b). However, it is important to note that the higher protection of cardioblasts was achieved with aqueous micellar dispersions, whereas the referent solutions were hydroalcoholic due to the necessity to dissolve the resveratrol. Resveratrol is a highly hydrophobic drug (Class II of the biopharmaceutical classification system) and its solubility is a limiting factor for absorption. Thus, the developed micellar system improved the solubility of resveratrol, which is a prerequisite for intracellular transport and protective action in cardioblasts.

**Figure 10:**
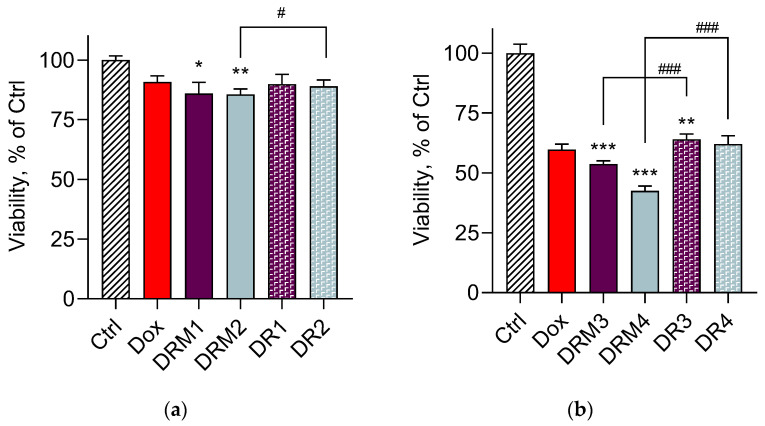
In vitro cytotoxic effect in lymphoma L5178 cells treated with the double-loaded doxorubicin/resveratrol micelles (DRM) and corresponding hydroalcoholic solutions of doxorubicin/resveratrol (DR). Ctrl—non-treated cells; DRM1 (double-loaded micelles) and DR1 (standard solution) containing 0.25 µM Dox and 1.3 µM Res (**a**); DRM2 (double-loaded micelles) and DR2 (standard solution) containing 0.25 µM Dox and 0.3 µM Res (**a**); DRM3 (double-loaded micelles) and DR3 (standard solution) containing 5 µM Dox and 26 µM Res (**b**); DRM4 (double-loaded micelles) and DR4 (standard solution) containing 5 µM Dox and 6 µM Res (**b**); * *p* < 0.05; ** *p* < 0.01; *** *p* < 0.001 vs. doxorubicin group; ^#^ *p* < 0.05; ^###^ *p* < 0.001 for double-loaded micelles vs. hydroalcoholic solutions.

The cell viability of lymphoma L5178 cells after treatment with the micellar system was evaluated in order to test whether double loading would retain the cytotoxic effect of doxorubicin on tumour cells. The double loading of resveratrol and 0.25 µM doxorubicin in micelles enhanced the cytotoxic effect, especially at the lower concentration of 0.3 µM resveratrol (DRM2) (Figure 10a). Moreover, the *t*-test showed a statistically significant difference between the groups of cells treated with the loaded micelles (DRM2) and the corresponding hydroalcoholic solution of both drugs (DR2). Doxorubicin at a concentration of 5 µM was more cytotoxic when loaded in micelles, which was more pronounced when combined with a lower concentration of resveratrol (6 µM) (Figure 10b). Furthermore, we observed statistically significant differences between the effects of the micellar dispersions and the corresponding hydroalcoholic solutions of non-encapsulated drugs. Thus, the simultaneous loading of resveratrol and doxorubicin in Pluronic micelles reduced the cardiotoxic effect of doxorubicin but did not decrease its cytotoxicity in tumour cells. This is a prerequisite for further investigations on cardioprotective and antiproliferative effects of the micelles, double-loaded with doxorubicin and resveratrol.

## 4. Conclusions

The developed double-loaded nanoscale micelles could be considered as a promising drug delivery system, since they provide a high loading of both drugs. Furthermore, the double-loaded micellar system could ensure the delivery of doxorubicin to tumour cells while lowering its toxicity on cardioblasts. Thus, the developed micelles made of a mixture of Pluronic P123 and F127 could serve as a useful tool for the development of novel pharmacotherapeutics.

## Figures and Tables

**Table 1:** Mean diameter, polydispersity index (PDI) and zeta potential of empty and double-loaded micelles.

Sample	Mean Diameter, nm	PDI	Zeta Potential, mV
Empty micelles (EM)	33 ± 2	0.291	−5.14
Double-loaded micelles (DRM)	26 ± 3	0.283	+4.15

**Table 2:** Kinetic parameters for release data fitted to the Korsmeyer–Peppas model.

Release Medium	Doxorubicin	Resveratrol
pH = 5.0	r^2^ = 0.9989 *n* = 0.497	r^2^ = 0.9396 *n* = 0.443
pH = 7.4	r^2^ = 0.9909 *n* = 0.530	r^2^ = 0.9749 *n* = 0.432

## Data Availability

Not applicable.
